# Clinical prediction models for patients undergoing total hip arthroplasty: an external validation based on a systematic review and the Dutch Arthroplasty Register

**DOI:** 10.2340/17453674.2024.42449

**Published:** 2024-11-25

**Authors:** Maartje BELT, Katrijn SMULDERS, B Willem SCHREURS, Gerjon HANNINK

**Affiliations:** 1 Research Department, Sint Maartenskliniek, Nijmegen; 2 Department of Orthopaedics, Radboud University Medical Center, Nijmegen; 3 Dutch Arthroplasty Register (Landelijke Registratie Orthopedische Interventies), ‘s-Hertogenbosch; 4 Department of Medical Imaging, Radboud university medical center, Nijmegen, The Netherlands

## Abstract

**Background and purpose:**

External validation is a crucial step after prediction model development. Despite increasing interest in prediction models, external validation is frequently overlooked. We aimed to evaluate whether joint registries can be utilized for external validation of prediction models, and whether published prediction models are valid for the Dutch population with a total hip arthroplasty.

**Methods:**

We identified prediction models developed in patients undergoing arthroplasty through a systematic literature search. Model variables were evaluated for availability in the Dutch Arthroplasty Registry (LROI). We assessed the model performance in terms of calibration and discrimination (area under the curve [AUC]). Furthermore, the models were updated and evaluated through intercept recalibration and logistic recalibration.

**Results:**

After assessing 54 papers, 19 were excluded for not describing a prediction model (n = 16) or focusing on non-TJA populations (n = 3), leaving 35 papers describing 44 prediction models. 90% (40/44) of the prediction models used outcomes or predictors missing in the LROI, such as diabetes, opioid use, and depression. 4 models could be externally validated on LROI data. The models’ discrimination ranged between poor and acceptable and was similar to that in the development cohort. The calibration of the models was insufficient. The model performance improved slightly after updating.

**Conclusion:**

External validation of the 4 models resulted in suboptimal predictive performance in the Dutch population, highlighting the importance of external validation studies.

Several prediction models have been developed for hip and knee arthroplasty, aiming to predict the probability of an outcome after surgery [1-6]. These predicted probabilities can provide valuable information to patients and clinicians as an aid in clinical decision-making and expectation management. However, existing prediction models for arthroplasty are often not suitable for use in clinical practice, due to either poor predictive performance or lack of external validation [7,8].

External validation plays an important role in assessing the generalizability and performance of these models in a different set of patients [9]. Ideally, data for external validation purposes is collected specifically for the purpose of external validation, but this approach can be time-consuming and resource-intensive. Another, more common option is to use previously collected data for external validation, although absence of variables or different variable definitions may complicate the use of existing databases.

Large datasets, such as (inter)national registries, are a potentially rich source for external validation. Registry data is relatively easily accessible and often includes large patient cohorts. However, one drawback is that registry data is not collected specifically for the purpose of external validation of prediction models. As a result, the definitions of predictor variables may differ from those required for external validation, or certain predictor variables may not be collected in the registry at all [10]. Nonetheless, it is worthwhile to explore whether joint registries can be utilized for external validation of clinical prediction models. The objective of this study was (i) to assess whether joint registries can be utilized for external validation of prediction models, and (ii) to evaluate whether published prediction models are valid for the Dutch total hip arthroplasty (THA) population.

## Methods

### Study design

The study was designed as a systematic literature search performed in PubMed from the date of inception to April 2023 for studies describing prediction models that predict the risk of revision or mortality after total joint arthroplasty (TJA).

The study was reported according to the Transparent Reporting of a multivariable prediction model for Individual Prognosis Or Diagnosis (TRIPOD) statement for prediction model studies [11].

### Use of joint registries for external validation

The search string was based on the keywords arthroplasty, prediction models, revision, and mortality as the latter 2 are outcomes available in the LROI (see Appendix A for the detailed search strategy). Literature was screened by 1 author (MB). Papers were excluded if no prediction model was described, or the model was not developed for TJA patients. To assess whether joint registries can be utilized for external validation of prediction models, we used the Dutch Arthroplasty Registry (LROI) as an example. We evaluated the utility of using joint registries for this purpose by evaluating the frequency of the predictors of the models that were found by the literature search and whether they are available in the LROI. Next, we evaluated the quality of the registry data by assessing the percentage of missing data per predictor variable, and whether the definitions of the variables used are standard or could be harmonized [10].

### External validity of published prediction models

Next, we selected prediction models from the literature search that could be externally validated on data from the LROI to test their validity in Dutch clinical practice. Models were included if a prediction model was developed for patients who underwent TJA, and when the outcome and all predictors in the model were available in the LROI. This resulted in 2 papers describing 4 prediction models (Table 1).

**Table 1:** 4 prediction models that were included from the literature

Paper	Model	Model coefficients ^a^
1 Venäläinen [3]	Logistic regression model predicting short-term revision (within 6 months) for dislocation	Linear predictor = –6.801 + 0.459_*_ASA class + 0.861_*_preoperative fracture + 0.675_*_previous contributing operations + 0.606_*_posterior surgical approach + 0.355_*_32-mm head diameter
2 Venäläinen [3]	Logistic regression model predicting short-term revision (within 6 months) for periprosthetic fracture	Linear predictor = –9.138 + 0.404_*_ASA class + 0.244_*_age (per 10 years) + 1.479_*_cementless fixation
3 Venäläinen [3]	Logistic regression model predicting short term mortality (within 6 months)	Linear predictor = –7.017 + 0.491_*_ASA class + 0.104_*_age (per 10 years) + 0.878_*_preoperative fracture
4 Paxton [2]	Logistic regression model predicting the risk of a revision surgery within 5 years after total hip arthroplasty	Linear predictor = –2.66834 – 0.01742_*_age + 0.215285_*_female sex + 0.067322_*_√BMI – 0.16622_*_osteoarthritis

a The predicted probability of the outcome is calculated as: 1/(1+e^(–(linear predictor)))

The 4 selected prediction models were all developed in patients undergoing primary THA. As a result, the study population was narrowed down from TJA to THA. 3 out of 4 identified models (models 1 to 3) were originally developed in a Finnish population [3]. Data on all primary THAs (n = 25,919) performed in Finland between May 2014 and January 2018 was collected in the Finnish Arthroplasty Register and used for model development. The first model (model 1) predicts the risk of short-term (< 6 months after primary THA) revision for dislocation. The second model (model 2) was developed to predict the risk of short-term (< 6 months after primary THA) revision for periprosthetic fracture. The third model (model 3) was developed to assess the risk of short-term (< 6 months) mortality after primary THA. The last model (model 4) aimed to predict the risk of revision within 5 years after primary THA (n = 20,592) [2]. It was developed on data from Kaiser Permanente’s Total Joint Replacement Registry. The cohort included all patients who had primary procedures performed between April 2001 and July 2008.

### LROI dataset for external validation

Data for external validation was obtained from the LROI, a nationwide population-based registry on TJAs performed in the Netherlands since 2007. All Dutch hospitals report patient characteristics, surgical techniques, prosthesis characteristics, and patient-reported outcomes of total joint arthroplasties to the LROI. The data completeness for primary total hip arthroplasties (THAs) was 97% in 2013 and up to 99% since 2016 [12].

### External validation cohorts

*Cohort 1.* For the validation of the first 3 models, the outcomes of interest were revision (models 1 and 2) or mortality (model 3) within 6 months after THA. Data on all registered primary THAs performed between January 2007 and December 2020 in the Netherlands was provided by the LROI. All surgeries before 2014 were excluded to match patient sampling time between the development and external validation cohort. Patients operated on after December 2019 were excluded to ensure sufficient follow-up time. Thus, we included all patients with a primary THA performed between January 2014 and December 2019 for the external validation.

*Cohort 2.* For the validation of the fourth model, a different group of patients was selected from the LROI dataset. As body mass index (BMI) was a predictor in the model, and BMI has only been registered in the LROI since 2014, all surgeries before 2014 were excluded. To ensure a minimum follow-up of 5 years, all arthroplasties performed after December 2015 were excluded. Hence, we included all patients who received a primary THA between January 2014 and December 2015.

### Predictor definitions LROI

The 4 models used a subset of the following predictors: sex, age, BMI, ASA classification, osteoarthritis or fracture as diagnosis for primary THA, the presence of 1 or more previous contributing surgeries, surgical approach (anterolateral or posterior), type of fixation (cemented or cementless), and head diameter (Table 1). All were reported to the LROI at the time of primary surgery. Osteoarthritis was defined as all types of osteoarthritis (including secondary arthritis and coxarthrosis). Fracture as diagnosis for primary THA was defined as the implantation of primary THA within 5 days after hip fracture (including medial/lateral collum fracture, femoral neck fracture, trochanteric femur fracture). Previous surgeries of the hip include: osteosynthesis, osteotomy, arthrodesis, Girdlestone procedure, arthroscopy, and/or other. Surgical approach was categorized as: straight lateral, posterolateral, anterolateral, anterior, straight superior, or other. An overview of the variable definitions of both the LROI and the model development papers can be found in Table 2. Two predictors had different definitions in the development paper compared with the LROI. In the development paper, surgical approach was categorized as posterior or anterolateral, where the LROI uses 6 categories. In the external validation, we used the posterolateral versus all other categories to calculate the predicted risk. Also, the predictor “previous surgeries” was defined slightly differently between the development paper and the LROI. Girdlestone procedure and arthroscopy are not explicitly mentioned as previous contributing surgery in the development paper but were included in the LROI data. Also, both included “other” as a category. In either case, it is not explicitly stated which operations are included, thus it is unclear whether the same previous surgeries are included in the predictor. In the external validation, we used the predictors as described above, according to the LROI definition.

**Table 2:** Overview of variable definitions in the development and validation cohorts

Variable	Definition
Mortality	
LROI	Retrieved from national insurance database
Model 1–3	Dates of death are retrieved from the Population Information System maintained by the Population Register Centre, Finland
Model 4	–
Revision	
LROI	Removal or exchange of the inlay, femoral head, acetabulum, and/or femur component
Model 1–3	Change or removal of at least 1 prosthetic component
Model 4	Removal or exchange of at least 1 prosthetic component
Reason for revision	
LROI	Infection; wear of cup/liner; periprosthetic fracture; malposition or malalignment; luxation; periarticular ossification; loosening of acetabular component; loosening of femur component; symptomatic metal on metal articulation; Girdlestone; other
Model 1–3	Dislocation or periprosthetic fracture of femur or acetabulum reported as main reasons for revision
Model 4	–
ASA	
LROI	I / II / III / IV
Model 1–3	I / II / III / IV
Model 4	–
Preoperative fracture	
LROI	Primary THA within 5 days after hip fracture (including medial/lateral collum fracture, femoral neck fracture, trochanteric femur fracture)
Model 1–3	Primary THA for fracture
Model 4	–
Previous surgeries	
LROI	Includes: osteosynthesis, osteotomy, arthrodesis, Girdlestone procedure, arthroscopy, and/or other
Model 1–3	Includes: osteotomy of acetabulum or femur, osteosynthesis of tibia or femur, or other (e.g., arthrodesis)
Model 4	–
Approach	
LROI	Straight lateral, posterolateral, anterolateral, anterior,straight superior, other
Model 1–3	Posterior, anterolateral (modified Hardinge)
Model 4	–
Head diameter	
LROI	22–28, 32, 36, and ≥ 38 mm
Model 1–3	28, 32, 36, and > 36 mm
Model 4	–
Age	
LROI	In years
Model 1–3	In years
Model 4	–
Type of fixation	
LROI	Cemented, cementless, hybrid: femur cemented, reverse hybrid: acetabulum cemented
Model 1–3	Cemented, cementless, hybrid, reverse hybrid
Model 4	–
Sex	
LROI	Male, female, non-specified, unknown
Model 1–3	Male, female
Model 4	Male, female
Osteoarthritis	
LROI	All types of osteoarthritis (including secondary arthritis and coxarthrosis) as primary diagnosis
Model 1–3	–
Model 4	Osteoarthritis as primary diagnosis

### Outcome definitions LROI

A revision surgery was defined as the removal or exchange of the inlay, femoral head, acetabulum, and/or femur component, and was registered in the LROI. In models 1 and 2, only revisions within 6 months for dislocation or for periprosthetic fracture were analyzed. Dislocation was defined as recurring dislocation of the hip prosthesis. Periprosthetic fracture was defined as a fracture around the hip prosthesis causing an interruption of the fixation or stability and therefore needing revision surgery. The reason for revision was reported by the surgeon directly after surgery to the LROI. In the fourth model, all revision surgeries within 5 years after primary THA were included as event. Model 4 and the LROI use the same definition for revision surgery. In the paper of models 1 to 3, the exact definition of the outcome was not described, and therefore the authors may have used another definition.

For model 3, the outcome of interest was mortality within 6 months after primary THA. Mortality is obtained from the Dutch national insurance database (Vektis), and linked to the LROI. Vektis contains records of all deaths of all Dutch citizens.

### Sample size

No formal sample size calculation was performed. All patients in the LROI who were eligible for the study were included. This resulted in validation cohorts that exceeded the development cohort and recommendations for sample size [13,14].

### Statistics

In cohort 1 (models 1 to 3), ASA was missing in 285 patients and age in 71 patients. In cohort 2, BMI was missing in 2,580 patients and age in 45 patients. Due to the low number of missing data points in LROI data (cohort 1: < 1%; cohort 2: < 5%), and assuming missing completely at random, we decided to do a complete case analysis. Patient age values were excluded if the age was above 105 years (n = 17) or below 10 years (n = 25). BMI values were excluded if BMI exceeded 70 (n = 29) or was below 10 (n = 2). These cut-off thresholds were applied according to LROI recommendations [15].

The baseline characteristics were described as means and standard deviation (SD) or median and interquartile range (IQR) for continuous variables (as appropriate), and number and percentage (%) of total for categorical variables.

To evaluate model performance on LROI data, we assessed discrimination and calibration. Discrimination of the models was assessed by calculating the area under the receiver-operating characteristic curve (AUC). The discrimination reflects the ability of a model to discriminate between those with and those without the outcome. For interpretation of AUC values, cut-off values < 0.7 (poor), 0.7–0.8 (acceptable), 0.8–0.9 (excellent), and > 0.9 (outstanding) were used [16]. Calibration was evaluated by plotting the observed probabilities against the predicted probabilities of the outcome and calculating the calibration slope and the intercept (or calibration-in-the-large) [17]. Calibration reflects the agreement between the predicted probability of developing the outcome as estimated by the model and the observed outcome. A perfect calibration-in-the-large (or mean calibration) has a slope of 1 and an intercept of 0. A calibration curve close to the diagonal indicates that the predicted probability corresponds well to the observed probability.

After the validation of the models on LROI data, models were updated in 2 steps [18]. First, the intercepts were recalibrated to improve calibration-in-the large by aligning observed outcome rates and mean predicted probability. Second, logistic recalibration was performed to correct miscalibration of the predicted probabilities, to prevent general over- or underestimation of risks. In this step, the model intercepts as well as the predictor coefficients were updated [18]. These updated models were re-evaluated by analyzing their discrimination and calibration performance.

All analyses were performed using R software (version 4.2.1; R Foundation for Statistical Computing, Vienna, Austria) with packages rms (v6.3.0) and CalibrationCurves (v1.0.0) [19-21].

### Ethics, funding, and disclosures

Data was made available by the LROI; however, restrictions apply to the availability of this data, which was used under license for the current study. All data was received completely de-identified. The LROI uses an opt-out system to require informed consent from patients. This study received no funding. No conflicts of interest were declared. Complete disclosure of interest forms according to ICMJE are available on the article page, doi: 10.2340/17453674.2024.42449

## Results

### Use of joint registries for external validation

**Figure 1:**
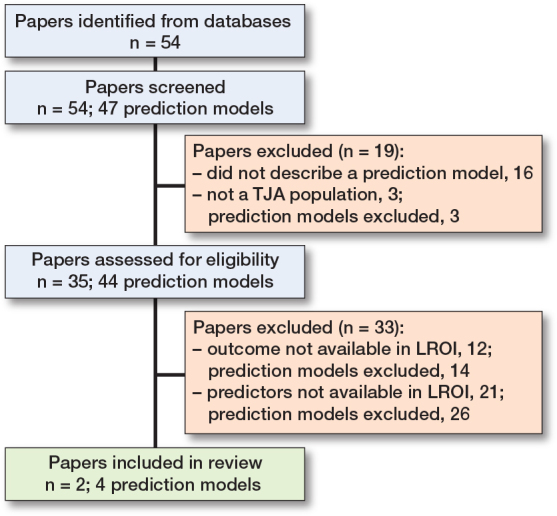
Flowchart of literature search.

Our literature search resulted in 54 hits, of which 16 papers did not describe a prediction model, and 3 papers described a non-TJA population, and were therefore excluded (Figure 1). This resulted in 35 papers describing 1 or more prediction models developed for a TJA population. A total of 44 unique prediction models were described in the 35 papers. While the literature search was aimed at outcomes that are available in the LROI, the prediction models also predicted outcomes other than revision or mortality. Complications, or specifically infection, was commonly used as outcome. A total of 193 unique predictors were used in the prediction models; only 31 occurred in more than 1 model (see Appendix B). The most prevalent predictors that are not available in the LROI are: diabetes mellitus (used in 9 prediction models), depression (used in 6 prediction models), insurance type (used in 6 prediction models), and opioid use (used in 6 prediction models). Most predictors that are available in the LROI have less than 1% missing data and are either measured in a standardized way or can be harmonized. BMI, which has been recorded since 2014, has a maximum of 4.6% missing data. This may limit the follow-up period for patients when BMI is used as a predictor, potentially impacting the validity of the results.

### External validity of published prediction models

Of the 35 papers from the literature search that described 44 prediction models, 14 models were excluded because the outcome of the model was not available in the LROI, and 26 models were excluded because the prediction models included predictors that were not available in the LROI (Figure 1; see Supplementary data for prediction model papers and reasons for exclusion). After excluding 40/44 models, 4 prediction models described in 2 papers were left [2,3]. Thus, 4 out of 44 models (9%) on a TJA population could be externally validated using LROI data.

### External validation cohort

*Cohort 1.* 178,422 patients received a primary THA between 2014 and 2020 in the Netherlands (Table 3). The mean age of the cohort was 69 years (SD 10.5), and 65% were female. Most patients received a THA due to osteoarthritis (87%). The baseline characteristics of the LROI validation cohort were comparable to the development cohort of models 1 to 3; only ASA and head diameter were differently distributed. The majority of the patients had ASA 2, while in the development cohort ASA 3–4 was more common. In the LROI, in 60% of the surgeries the head diameter was 32 mm, compared with the development cohort where 73% had 36 mm heads. Comparing the outcome prevalence between the cohort on which the models were developed and the LROI validation cohort revealed a prevalence of revision within 6 months due to dislocation of 0.4% in the LROI, and 0.7% in the development cohort (Table 3). A revision due to fracture within 6 months occurred in 0.3% of the patients in the LROI, and 0.5% in the development cohort. The prevalence of mortality < 6 months was 0.6% in the LROI, and 0.7% in the development cohort.

**Table 3:** Baseline characteristics. Values are count (%) unless otherwise specified

Factor	External validation		Development study model 1–3 (n = 8,640)
	cohort 1 (LROI) (n = 178,422)	cohort 2 (LROI) (n = 56,675)	
Female sex	116,198 (65)	37,132 (66)	4,967 (58)
Age mean (SD)	68.9 (10.5)	68.8 (10.6)	67.6 (10.8)
BMI mean (SD)	27.3 (4.6)	27.3 (4.6)	28.1 (4.8)
missing	3,135 (1.8)	2,580 (4.6)	971 (11)
ASA			
1	30,832 (17)	11,183 (20)	1,014 (12)
2	114,124 (64)	36,832 (65)	4,065 (48)
3–4	33,181 (19)	8,401 (15)	3,357 (40)
missing	285 (0.2)	259 (0.5)	204 (2.4)
Diagnosis			
osteoarthritis	154,597 (87)	48,942 (86)	7,138 (86)
fracture	7,918 (4.4)	1,128 (2.0)	527 (6.4)
inflammatory arthritis	195 (0.1)	66 (0.1)	144 (1.7)
missing	482 (0.3)	377 (0.7)	321 (3.7)
Previous surgeries	8,421 (4.7)	2,710 (4.8)	167 (1.9)
Approach			
posterolateral	102,677 (58)	34,605 (61)	6,731 (80)
anterior	44,044 (25)	8,261 (15)	–
anterolateral	8,330 (4.7)	2,948 (5.2)	1,688 (20)
straight lateral	21,626 (12)	10,477 (19)	–
other	1,437 (0.8)	154 (0.2)	–
missing	308 (0.2)	230 (0.4)	221 (2.5)
Head diameter, mm			
22–28	32,736 (18)	14,204 (25)	105 (1.2)
32	106,498 (60)	30,547 (54)	2,076 (25)
36	36,775 (21)	11,406 (20)	6,130 (73)
≥ 38	513 (0.3)	143 (0.3)	89 (1.1)
missing	1,905 (1.1)	115 (0.2)	240 (2.7)
Type of fixation			
cementless	115,017 (65)	35,387 (62)	5,448 (65)
cemented	44,727 (25)	15,172 (27)	676 (8.1)
hybrid	18,527 (10)	6,005 (11)	2,205 (27)
missing	151 (0.1)	111 (0.2)	311 (3.5)
Revision for			
dislocation < 6 months	(0.4)	–	(0.7)
fracture < 6 months	(0.3)	–	(0.5)
Mortality < 6 months	(0.6)	–	(0.7)
Revision < 5 years	–	(3.1)	–

NB. The baseline characteristics of the test cohort of model 4 were not described in the article, and thus not included in this table.

*Cohort 2.* 56,675 patients received a primary THA between 2014 and 2015. The baseline characteristics were comparable to the patients operated on between 2014 and 2020. The baseline characteristics of the development cohort of model 4 were not described in the development paper, and thus could not be included (Table 3). The prevalence of revision < 5 years was 3.1% in the LROI, and 3.1% in the development cohort.

### External validation

Model 1, predicting the risk of revision for dislocation < 6 months, had a poor discriminative ability; the AUC was 0.64 (CI 0.59–0.68) in the external validation cohort (Table 4). The AUC of model 2, which predicts risk of revision for fracture < 6 months, was 0.67 (CI 0.65–0.70). Model 3, which predicts the risk for mortality < 6 months, had the best discriminative ability of the 4 models; with an AUC of 0.79 (CI 0.77–0.80) the discrimination was acceptable. The lowest discrimination was that of model 4, predicting risk of all-cause revision within 5 years, with an AUC of 0.53 (CI 0.51–0.54). Discriminative ability of the models in the external validation cohort was similar to the discriminative ability in the development cohorts (Table 4).

**Table 4:** Area under the curve (AUC) with 95% confidence intervals for the development cohort and in the LROI dataset

Model	AUC external validation cohort	AUC test cohort development study
1. Revision for dislocation < 6 months	0.64 (0.59–0.68)	0.64 (0.56–0.72)
2. Revision for fracture < 6 months	0.67 (0.65–0.70)	0.65 (0.58–0.72)
3. Mortality < 6 months	0.79 (0.77-0.80)	0.84 (0.78–0.90)
4. Revision < 5 years	0.53 (0.51–0.54)	0.56 ^a^

a The AUC of model 4 was not described in the paper but may be requested from the authors.

**Figure 2:**
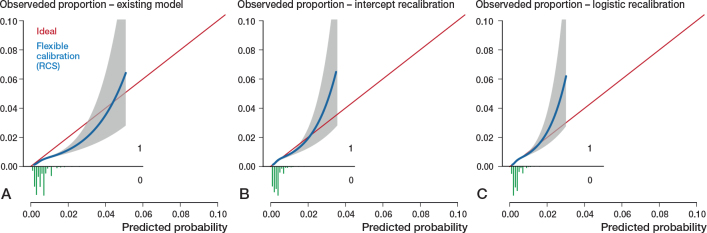
Calibration plots external validation of model 1. Discrimination, c-statistics: 0.64 (0.59–0.68). A. Calibration plot for predicted risk of revision for dislocation within 6 months after THA based on the existing model, externally validated on LROI data. The calibration curve allows for examination of calibration across a range of predicted values. A curve close to the diagonal line (i.e., perfect calibration) indicates that predicted (x-axis) and observed probabilities (y-axis) correspond well. The linear bar chart shows the distribution of patients with (= 1) or without (= 0) an observed outcome. Calibration, intercept: –0.39 (–0.46 to –0.31), slope: 0.92 (0.79–1.06). B. Calibration plot after intercept recalibration. Calibration, intercept: –0.00 (–0.07 to 0.07), slope: 0.92 (0.79–1.06). C. Calibration plot logistic recalibration. Calibration, intercept: 0.00 (–0.07 to 0.07), slope: 1.00 (0.85–1.15.

**Figure 3:**
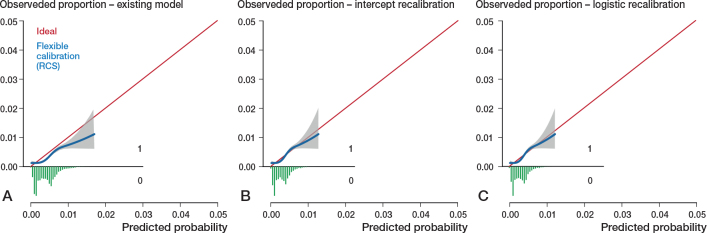
Calibration plots external validation of model 2. Discrimination, c-statistics: 0.68 (0.65–0.70). A. Calibration plot for predicted risk of revision for periprosthetic fracture within 6 months after THA based on the existing model, externally validated on LROI data. See Legend to Figure 2. Calibration, intercept: –0.30 (–0.38 to –0.21), slope: 0.97 (0.82–1.11). B. Calibration plot after intercept recalibration. Calibration, intercept: –0.00 (–0.08 to 0.08), slope: 0.97 (0.82–1.11). C. Calibration plot after logistic recalibration. Calibration, intercept: –0.00 (–0.08 to 0.08), slope: 1.00 (0.85–1.15).

**Figure 4:**
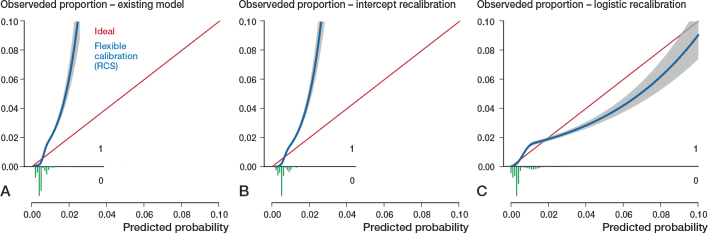
Calibration plots external validation of model 3. Discrimination, c-statistics: 0.79 (0.77–0.80). A. Calibration plot for predicted risk of mortality within 6 months after THA based on the existing model, externally validated on LROI data. See Legend to Figure 2. Calibration, intercept: 0.12 (0.06 to 0.18), slope: 2.24 (2.12–2.36). B. Calibration plot after intercept recalibration. Calibration, intercept: –0.00 (–0.06 to 0.06), slope: 2.24 (2.12–2.36). C. Calibration plot after logistic recalibration. Calibration, intercept: –0.00 (–0.06 to 0.06), slope: 1.00 (0.95–1.05).

**Figure 5:**
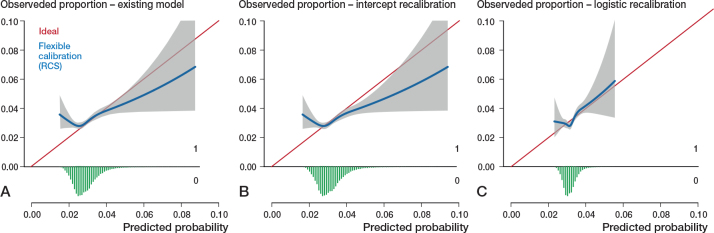
Calibration plots external validation of model 4. Discrimination, c-statistics: 0.53 (0.52–0.54). A. Calibration plot for predicted risk of revision within 5 years after THA based on the existing model, externally validated on LROI data. See Legend to Figure 2. Calibration, intercept: 0.08 (0.03 to 0.12), slope: 0.50 (0.29–0.71) B. Calibration plot for predicted risk of revision within 5 years after THA after intercept recalibration. Calibration, intercept: –0.00 (–0.05 to 0.05), slope: 0.50 (0.29–0.71). C. Calibration plot for predicted risk of revision within 5 years after THA after logistic recalibration. Calibration, intercept: –0.00 (–0.05 to 0.05), slope: 1.00 (0.57–1.43).

All models had far from optimal calibrated risk predictions (Figures 2A–5A). Model 3 largely underestimated the risk of mortality within 6 months. Predicted probabilities between 1% and 2.5% were lower than observed proportions. The other 3 models generally overestimated the risk of revision. The intercept and slope are included in the calibration plot. Calibration plots were not presented in the development papers, and therefore could not be compared.

### Model updating

Calibration of all 4 models improved slightly by recalibrating the intercept. Logistic recalibration improved the calibration of all models (Figures 2C–5C). In model 3, the underestimation of probabilities improved to a slight overestimation of the predicted risks above 2%. The calibration of the other models improved to a lesser extent, although the predicted risks were, overall, more accurate. In model 1, the logistic recalibrated model accurately predicted risks below 2%. In model 2, the logistic recalibrated model accurately predicted risks below 1%. The logistic recalibrated model 4 accurately predicted risks between 3% and 4%. The discrimination of the models did not improve after updating.

## Discussion

In this study, we assessed whether joint registries can be utilized for external validation of prediction models, and we evaluated the performance of 4 published prediction models in Dutch clinical practice using data from the LROI. We showed that registry data can be used for external validation; however, the use of registry data for external validation is heavily reliant on the availability of predictors and outcomes in the registry. The predictors that are available in the LROI seem to have sufficient completeness to be used for external validation. The discrimination in the validation cohorts was similar to the discrimination in the development cohorts. Although the models tended to over- or underestimate risks at higher predicted probabilities, they demonstrated good calibration and outperformed individual risk factors at lower predicted probabilities, which cover the majority of the data. However, due to unavailability of calibration plots of the models on the development cohort, a comparison between development and validation cohorts could not be made.

Our results support the feasibility of use of registry data for external validation of prediction models. A systematic review by Groot et al. showed that only 10/59 of the available machine learning prediction models for orthopedic surgical outcome were externally validated [22]. These 10 models were externally validated in 18 different studies. However, only 2 studies used registry data for external validation. The other studies did use existing data sets, which were collected in a single institution in the majority of the studies (14/18 studies). Furthermore, another study in arthroplasty patients also used registry data for prediction model development. Garland et al. used data from 2 nationwide registries to develop and externally validate a prediction model for 90-day mortality after THA [1]. These results, together with the current study, show that for future external validation studies the use of national registries is possible and worth considering.

The critical factor for the use of registry data for external validation is the availability of variables in registries. Of the 35 papers describing prediction models in our literature search, only 2 papers described models that could be validated using LROI data. This was due to the unavailability of predictors (e.g., diabetes mellitus or other comorbidities) or the unavailability of the outcome (e.g., infection rate, adverse events). Previous studies aiming to externally validate models using a specific registry also reported limitations as result of variable unavailability [23-25]. Slieker et al. (2021) aimed to externally validate models for nephropathy in patient with diabetes mellitus type 2 [23]. In this study, only 25% of prediction models were excluded due to unavailability of prediction or outcome variables. Hueting et al. aimed to validate models for breast cancer patients in the Netherlands Cancer Registry (NCR) [24]. More in line with our results, 78% of the models were excluded due to variable unavailability. The limited availability of variables can be explained by the aim of registries to monitor and compare prostheses, and the need to limit the administrative burden. Conversely, these results can also indicate that important variables are lacking in a registry when the variables show strong predictive value in multiple prediction models. In addition, all models in this study were also developed on registry data, and thus were presumably also based on a limited number of available variables. Because registries are designed to monitor prosthesis designs, the available variables do not necessarily have the strongest association possible with the outcome of interest, which may have affected the predictive performance of the models.

The included prediction models performed suboptimally in the Dutch THA population. The discriminative ability was insufficient in 3 out of 4 models. In addition, the calibration plots provide a visual interpretation of how well predicted probabilities align with observed probabilities across the range of predictions. The models provided well-calibrated probabilities within a narrow range of predicted probabilities. For example, the model predicting revision for dislocation within 6 months accurately predicted risks below 2%. However, within the lower well-calibrated range, it is unlikely that a patient and surgeon jointly would decide to refrain from surgery based on this prediction. A good calibration in higher ranges of probabilities is therefore important as this may affect decision-making. Therefore, understanding model performance in practice is crucial, as poorly calibrated prediction models can result in incorrect and potentially harmful clinical decisions [16]. Even if a model appears to be well calibrated and shows good discrimination, this does not necessarily imply it will have added benefit in clinical practice [26].

Models with poor performance are not easily improved. One factor affecting a model’s predictive ability is a different prevalence of the outcome in development and validation cohorts. To minimize this effect, the model can be recalibrated by adjusting the intercept or through logistic recalibration. Logistic recalibration refers to the updating of the original regression coefficients with new data to adjust the equation to local and contemporary circumstances [27,28]. Recalibration can be particularly useful to correct miscalibration of the predicted probabilities, when there is general over- or underestimation of risks.

In our study, ASA score was distributed differently in the LROI data set compared with the development cohort. This discrepancy may be explained by differences in background morbidity, variations in access to surgery, and scoring differences [29]. The difference in ASA distribution may have prevented perfect calibration of LROI data, even after applying recalibration. Besides intercept updating and logistic recalibration, other updating methods are available to improve existing prediction models to better suit other populations. These methods include adding more predictors and/or re-estimating predictor coefficients [17]. Opinions on whether model updating is appropriate in external validation differ among researchers [9]. Some argue that changing or adding predictors is essentially constructing a new prediction model, which in turn requires internal and external validation. Furthermore, it can also be questioned whether extending an existing model to improve poor performance is favorable over developing an entirely new model. Nonetheless, even if models’ performance would have been good, clinical utility is not guaranteed and remains to be investigated in clinical impact evaluation [27].

### Limitations

The definitions of some predictors differed between the data sets underlying the development and external validation models. The definition of type of fixation and approach were not identical, which may have affected the model performance [30]. Harmonization of variables and definitions across joint registries is currently an important topic [31,32], which will positively influence the feasibility of using registry data for validation of models in different countries. Other factors that may affect predictive performance and may limit generalizability of prediction models to other settings are differences in healthcare systems, time period in which patients were treated, and differing treatment strategies between countries, for example, differences in THA approach or in the preferred type of fixation [33].

### Conclusion

Registry data can be used for external validation of prediction models, although it is heavily reliant on the availability of predictors and outcomes in the registry. External validation of the 4 models resulted in suboptimal predictive performance in the Dutch population.

*In perspective,* prediction models should be externally validated to assess their performance in new settings before they are implemented in clinical practice, in order to prevent incorrect predictions. To strengthen the utility of registry data for future prediction models, efforts could focus on incorporating additional relevant predictors and outcomes within registries. This will improve both model development and external validation efforts and help refine predictive accuracy.

### Supplementary data

Search strategy and a list of excluded prediction model papers are available as supplementary data on the article page, doi: 10.2340/17453674.2024.42449

## Supplementary Material



## Notes

MB, KS, WS, GH: concept and design. MB: Collection and assembly of data. MB, GH: data analysis. MB, KS, WS, GH: interpretation of the data. MB: drafting of manuscript. MB, KS, WS, GH: critical revision and final approval of the version submitted.

Handling co-editors: Keijo Mäkelä and Robin Christensen

*Acta* thanks Marina Torre and Mikko S Venäläinen for help with peer review of this manuscript.
