# Supplementary material for: Clinical prediction models for patients undergoing total hip arthroplasty: an external validation based on a systematic review and the Dutch Arthroplasty Register
Source: Acta Orthop. 2024 Nov 25;95:685–94. doi: 10.2340/17453674.2024.42449 (PMC11587164; doi:10.2340/17453674.2024.42449)
Supplement: Supplementary file 1 [file ActaO-95-42449-s1.pdf]

Supplementary data

A. Literature search

PubMed search (last search April 12th 2023) strategy:

(prediction [title] OR prognostic model [title] OR risk calculator* [title] OR risk model [title] OR nomogram [title] OR risk score [title])

AND arthroplast* [tiab] AND (hip [tiab] OR knee [tiab])

AND (revision [tiab] OR failure [tiab] OR reoperation [tiab] OR mortality [tiab])

B. Literature search results

Our literature search resulted in 54 hits, of which 15 papers did not include a prediction model, and 4 papers described a different population. These papers were therefore excluded from this overview. The remaining papers are listed below. The papers highlighted in blue are included in our proposal and can be externally validated using Dutch Arthroplasty Register data.

First author	Year	Cohort	Outcome	Predictors	Sample size	DOI	Reason
**Buddhiraju**	2023	revision TKA	nonhome discharge	age, BMI, revision for infection, perioperative transfusion, ASA	52,533	10.1016/j.arth.2023.02.054	Outcome + predictors not available in LROI
**Klemt**	2023	revision TKA	PJI	smoking, age, gender, medicare insurance, revision indication, depression, diabetes, extremity condition, obesity, drug abuse, >1 open procedure prior to revision TKA	1,432	10.1055/s-0043-1761259	not all predictors available in LROI

**Cuthbert**	2022	THA and TKA	time to revision	THA: femoral cement, patient depression, use of pain medication (opioids), gastro-oesophageal refux disease, gender, age and steroid responsive diseases<br>TKA: age, use of pain medication (opioids), use of patella resurfacing, prosthesis stability, prosthesis bearing surface, and patient depression	321,945 + 151,113	10.1186/s12874-022-01644-3	not all predictors available in LROI
**Klemt**	2022	THA	revision within 2 years	charlson comorbidity index, obesity, depression, nonprivate insurance, diabetes, age, gender	7,397	10.5435/JAAOS-D-21-01039	not all predictors available in LROI
**Lu**	2022	THA	1 year mortality	age, Charlson Comorbidity Index, ASA, urea, serum Ca2+, postoperative hemoglobin	246	10.3389/fsurg.2022.926745	not all predictors available in LROI
**Onishchenko**	2022	THA and TKA	Postoperative major adverse cardiac events	comorbidities: respiratory, cardiovascular, opthalmological, hypertensive, immune, hematologic, reproductive, central nervous system, all infections, injuries, respiratory infections, musculoskeletal, frailty, integumentary, metabolic, endocrine, digestive, psychiatric, bacterial infections, peripheral nervous system, fungal & infections, neoplasms, otic, allergies, developmental.	445,391	10.1161/JAHA.121.023745	not all predictors available in LROI
**Pakarinen**	2022	THA	revision for dislocation	mean corpuscular volume, age, gender, Charlson comorbidity index, use of anti-parkinson drugs, pyychiatric or neurological disease, femoral head size, serum creatinine level, ASA, BMI, acetabular fixation, primary reason for surgery, use of antiepileptic drugs, femoral fixation	16,454	10.1371/journal.pone.0274384	not all predictors available in LROI
**Sancho**	2022	DAIR	failed DAIR	serum CRP levels, positive blood cultures, indication for index arthroplasty other than osteoarthritis, not exchanging the modular components, use of	64	10.3390/diagnostic	Outcome + predictors not

				immunosuppressive medication, late acute (haematogenous) infections, methicillin-resistant Staphylococcus aureus infection, overlying skin infection, polymicrobial infection, and age		s1209209 7	available in LROI
**Wyles**	2022	THA	dislocation	age, gender, BMI, neurologic disease, spine disease, spine procedure, THA indication, approach/liner/headsize	29,349	10.2106/J BJS.21.01 171	not all predictors available in LROI
**Xie**	2022	TKA	90 day local complication s	reoperation (including implant revision or removal for any reason and manipulation under anesthesia), infection, bleeding requiring ≥4 unit transfusion of red blood cells within 72 hours of surgery, and peripheral nerve injury	410	10.1177/ 23259671 21107333 1	Outcome + predictors not available in LROI
**Andersen**	2021	TKA	2 year revision	gender, BMI, age, pain rest, pain activity, diabetes mellitus, previous surgery in the knee, OKS, EQ-5D, length of stay, duration of surgery, number of comorbiditites	538	10.1016/j. jor.2021.0 3.001	not all predictors available in LROI
**Fassihi**	2021	THA	30 day mortality	preoperative international normalized ratio, age, body mass index, operative time, and preoperative hematocrit	77,145	10.1016/j. jor.2021.1 1.013	not all predictors available in LROI
**Garland**	2021	THA	90 day mortality	Age, sex, ASA, cancer, CNS disease, kidney disease, obesity	53,099	10.1302/ 0301- 620X.103 B3.BJJ- 2020- 1249.R1	not all predictors available in LROI
**Klemt**	2021	revision TJA for PJI	reinfection	previous DAIR, previous surgeries, obesity, drug abuse, depression, smoking, presence of Enterococcus species	1081	10.1016/j. arth.2020 .08.004	Outcome + predictors not available in LROI

**Meyer**	2021	THA and TKA	adverse events	hospital frailty score, operative time, gender, age, ASA	565	10.1007/s00264-021-05038-w	Outcome + predictors not available in LROI
**Shah**	2021	THA	complications	Malnutrition, dementia, cancer, COPD, medicare, chronic arthrosclerosis, renal failure, other insurance, osteoarthritis, workers' compensation, skeletal disorders, Medi-Cal, rheumatoid arthritis, diabetes, morbid obesity, hospital volume, age	545	10.1016/j.arth.2020.12.040	Outcome + predictors not available in LROI
**Venäläinen**	2021	THA	Short-term revision for infection, revision for dislocation, revision for fracture, death	Infection: ASA, gender, BMI, anesthesia<br>Dislocation: ASA, pre-op diagnosis, previous op, surgical approach, head diameter<br>Fracture: ASA, age, fixation<br>Death: ASA, age, preop diagnosis	25,919	10.2106/JBJS.OA.20.00091	included
**Williams**	2021	TKA	90 day mortality	age, gender, cancer, COPD, gout, heart failure, hypertension, kidney disease, osteoarthritis, diabetes mellitus type 2, opioid use, psycholeptics use	193,615	10.1007/s00167-021-06799-y	not all predictors available in LROI
**Dibra**	2020	THA and TKA	discharge destination	age, gender, ambulation, walking aids, use of community support, postoperative caregiver,	716	10.1016/j.arth.2020.05.057	Outcome + predictors not available in LROI
**Meyer**	2020	THA and TKA	adverse events	Hospital Frailty Risk Score, age, sex, ASA	8250	10.1016/j.arth.2020.06.087	Outcome + predictors not available in LROI

**Zhang**	2020	TKA	Megaprosthetic failure	motion mode (fixed/hinged), BMI, type of surgery (primary/revision), type of prosthesis, length of bone resection, operative time	214	10.1016/j.arth.2020.05.016	not all predictors available in LROI
**Harris**	2019	TKA and THA	30 day mortality	Age, ASA, functional health status, bleeding disorders, dialysis, disseminated cancer, sepsis, >10% loss body weight	107,792	10.1097/CORR.00000000000000601	not all predictors available in LROI
**Jain**	2019	THA, TKA, and posterior lumbar fusions	90 day complications, 90 day readmission, and 1 year revision surgery	pre-operative opioid use	14,734 + 32,667 + 10,681	10.2106/JBJS.18.00502	Outcome + predictors not available in LROI
**Klausing**	2019	TKA	postoperative medical complications	Charlson Comorbidity Index, Index of Coexistent Disease, age, hemoglobin, hematocrit, creatinine, leukocytes, c-reactive protein, international normalized ratio, partial thromboplastin time.	649	doi: 10.1016/j.arth.2018.12.034	Outcome + predictors not available in LROI
**Verbeek**	2019	TKA	5 year functional outcome	Age, gender, functional KSS, reason for revision, type of bone defect	295	10.1007/s00167-019-05365-x	Outcome + predictors not available in LROI
**Harris**	2018	TKA and THA in veterans	30 day mortality	Age, CVA, PTCA, dyspnea-minimal exertion, dyspnea rest, albumin, thromboplastin time, wound infection, dementia, ulcers, malignancy, hemiplegia, angina, PVD, ASA	70,569	10.1016/j.arth.2017.12.003	not all predictors available in LROI
**Starr**	2018	TKA	revision TKA	Chronic opioid use, age, gender, BMI, diabetes, chronic kidney disease, nonchronic opioid use	32,297	10.1097/AJP.00000000000000544	not all predictors available in LROI

**Tan**	2018	THA and TKA	Periprosthetic joint infection	BMI, gender, government insurance, tha/tka/revision, prior procedures, comorbidities, smoking, drug abuse	27,717	10.2106/JBJS.16.01435	not all predictors available in LROI
**Everhart**	2016	TKA, revision TKA, THA, revision THA	30 day infection, 1 year infection	Procedure, COPD, Diabetes mellitus, rheumatoid arthritis, smoking, osteomyelitis, fracture, morbid obesity, bone cancer, reaction to implant, staphylococcal septicemia,	6,789	10.2106/JBJS.15.00988	not all predictors available in LROI
**Hussey**	2016	Metal-on-metal THA	Revision risk	Harris hip score, blood metal ion levels	1,709	10.2106/JBJS.15.00685	not all predictors available in LROI
**Inacio**	2015	THA and TKA	Revision surgery	Age, gender, diagnosis (ICD-10), Charlson, ATC code comorbidities, Elixhauser	11,848 + 18,972	10.1016/j.arth.2015.06.009	not all predictors available in LROI
**Paxton**	2015	THA and TKA	Revision risk	TKA: Age, sex, BMI, Diabetes mellitus, osteoarthritis, post traumatic artritis, osteonecrose<br>THA: sex, age, BMI, osteoarthritis	22,721 + 41,750	10.1007/s11999-015-4506-4	included
**Sabry**	2014	TKA	infection after two-stage revision	BMI, time from index surgery, duration of symptoms, number of previous surgeries, hemoglobin, soft tissue coverage required, previous infection same joint, previous two-stage revision, type of organism, diabetes, immunocompromised, heart disease	314	10.1016/j.arth.2013.04.016	not all predictors available in LROI
**Wuerz**	2014	THA and TKA	major postoperative complications	Lowest heart rate, extimated bloos loss, blood urea nitrogen, type of arthroplasty (primary/partial/revision), ethnicity, ASA, comorbidities, fracture	3511	10.1016/j.arth.2013.09.007	outcome not available in LROI

**Bozic**	2013	THA	2 year PJI and 90 day mortality	PJI: Gender, age, alcohol abuse, depression, electrolyte disorder, peptic ulcer disease, urinary tract infection, rheumatologic disease, preoperative anemia, cardiopulmonary (cardiac arrhythmia, congestive heart failure, ischemic heart disease, chronic pulmonary disease) comorbidities, and peripheral vascular disease.<br>Mortality: gender, age, electrolyte disorder, hemiplegia/paraplegia, hypertension, hypothyroidism, metastatic tumor, preoperative anemia, coagulopathy, cardiopulmonary (congestive heart failure, chronic pulmonary disease) and psychiatric (psychoses, depression) comorbidities, malignancies, and peripheral vascular disease	53,252	10.1007/s11999-012-2605-z	not all predictors available in LROI
